# Electrical Microneedles for Wound Treatment

**DOI:** 10.1002/advs.202409519

**Published:** 2024-11-08

**Authors:** Yu Wang, Lijun Cai, Lu Fan, Li Wang, Feika Bian, Weijian Sun, Yuanjin Zhao

**Affiliations:** ^1^ Department of Rheumatology and Immunology Nanjing Drum Tower Hospital School of Biological Science and Medical Engineering Southeast University Nanjing 210096 China; ^2^ Wenzhou Institute University of Chinese Academy of Sciences Wenzhou 325001 China; ^3^ Department of Gastrointestinal Surgery The First Affiliated Hospital Wenzhou Medical University Wenzhou 325035 China; ^4^ Shenzhen Research Institute Southeast University Shenzhen 518071 China

**Keywords:** electrical, electrical stimulation, mechanism, microneedles, wound healing

## Abstract

Electrical stimulation has been hotpot research and provoked extensive interest in a broad application such as wound closure, tissue injury repair, and nerve engineering. In particular, immense efforts have been dedicated to developing electrical microneedles, which demonstrate unique features in terms of controllable drug release, real‐time monitoring, and therapy, thus greatly accelerating the process of wound healing. Here, a review of state‐of‐art research concerning electrical microneedles applied for wound treatment is presented. After a comprehensive analysis of the mechanisms of electrical stimulation on wound healing, the derived three types of electrical microneedles are clarified and summarized. Further, their applications in wound healing are highlighted. Finally, current perspectives and directions for the development of future electrical microneedles in improving wound healing are addressed.

## Graphical abstract

A review on state‐of‐art research concerning electrical microneedles applied for wound treatment is presented. After a comprehensive analysis of mechanisms of electrical stimulation on wound healing, the derived three types of electrical microneedles are clarified and summarized. Further, their applications in wound healing are highlighted. Finally, current perspectives and directions for the development of future electrical microneedles in improving wound healing are addressed.

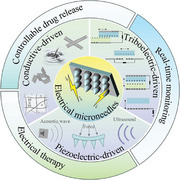

## 1 Introduction

Skin, the first line of the human body's defense, is responsible for protecting tissues and internal organs, thermoregulation, and resisting external aggression.^[^ ^1^, ^2^, ^3^, ^4^, ^5^ ^]^ Because of the high prevalence of skin damage caused by trauma, burns, chronic diseases, and other external hazards, wound therapy has become a globally concerning issue, posing an increased burden to the economy and society.^[^ ^6^, ^7^, ^8^, ^9^, ^10^, ^11^ ^]^ As the biological process of wound healing is complex, which mainly regards hemostasis, inflammation, proliferation, and remodeling, traditional treatment approaches like debridement and dressings are insufficient, and thus great urgent calls for effective and powerful wound therapy protocols.^[^ ^12^, ^13^, ^14^, ^15^, ^16^, ^17^ ^]^ Given this consideration, extensive endeavors have been concentrated on modulating physiological and immune processes during wound recovery. Notably, it has been proved that bioelectricity plays a crucial role in regulating cell behaviors including cell migration, proliferation, and differentiation, which shows positive effects on wound closure, tissue injury repair, nerve regeneration, etc.^[^ ^18^, ^19^, ^20^, ^21^, ^22^ ^]^ Taking inspiration from this, electrical stimulation has been widely explored and considered as one of the attractive approaches for wound treatment.^[^ ^23^, ^24^, ^25^ ^]^ Although achieved many progresses, in‐depth understanding of the mechanisms of electrical stimulation on wound healing has not been adequately discussed.

Driven by the advancement of material science and micro/nanofabrication technologies, microneedles have emerged and received growing attention in therapeutic and diagnostic medical applications.^[^ ^26^, ^27^, ^28^, ^29^ ^]^ Benefitting from minimal invasiveness, pain‐free, and self‐administration feasibility, microneedles, an array of micrometer‐scale needles, show great promise in acting as transdermal delivery systems for the dermis permeation of diverse therapeutic agents involving various macromolecules (proteins, genes or vaccines) and small molecule drugs.^[^ ^30^, ^31^, ^32^, ^33^ ^]^ Especially, some stimuli‐responsive elements such as light, ultrasound, magnetic field, electric field, etc. can be integrated with microneedles for achieving controllable drug release, thereby enhancing the therapeutic effectiveness.^[^ ^34^, ^35^, ^36^, ^37^, ^38^ ^]^ Among them, attributed to the great potential of the electrical field on wound healing, electrical microneedles exhibit appealing superiority. Thanks to the minimal invasiveness of microneedles, the enhanced penetration depth contributes to creating transdermal pathways and obtaining higher electric field density, thus improving therapeutic efficacy. Up to date, a variety of electrical microneedles have been developed.^[^ ^39^, ^40^, ^41^, ^42^ ^]^ According to their working mechanisms, electrical microneedles can be classified into three types conductive‐driven, triboelectric‐driven, and piezoelectric‐driven microneedles. Due to the outstanding merits of safety, well‐tolerated, and highly cost‐effective, these electrical microneedles have shown inspiring progress in wound treatment, especially from the perspectives of controllable drug release, real‐time monitoring, and electrical therapy. Despite these successes, there are few reviews summarizing these electrical microneedles and focusing on discussing the role of these electrical microneedles in wound healing.

**Figure 1:**
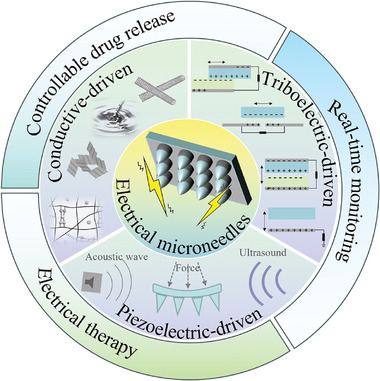
An overview of electrical microneedles for wound treatment.

In this review, the potential of electrical stimulation exerted on wounds is investigated and we will give a comprehensive review of the latest advancement of electrical microneedles (**Figure** **1**). Specifically, we start with some basic concepts to provide a distinct understanding of endogenous electric field, followed by the mechanisms of electrical stimulation on wound healing. Recent progress in three types of electrical microneedles is illustrated, including the integration manners between microneedles and electrical signals, and their functional differences. Subsequently, emphasis is given to the introduction of the applications of electrical microneedles in wound therapy. Finally, the remaining challenges and future opportunities of electrical microneedles for wound treatment are also analyzed. We hope this review can help readers gain further insights into the electrical microneedles, and steer the current efforts to promote the clinical advancement of wound disease treatment.

## 2 Mechanisms of Electrical Stimulation on Wound Healing

Undamaged skins have an electrical potential difference upon their epithelium of about 10–60 mV, while once skin is damaged, the electrical potential will be dramatically disordered and increased to 100–150 mV mm^−1^.^[^ ^43^ ^]^ Such discovery has inspired scientists to explore the relationship between the electrical potential and the healing process of wounds. In the past decade, the research on endogenous electric field has unraveled the veil of electrical stimulation to accelerate wound healing, which demonstrates that endogenous electric field has a positive impact on cell migration and proliferation.^[^ ^44^, ^45^ ^]^ Motivated by this, attention has turned to an exogenous electric field. Up to date, great progresses has been acquired on the exploration of external electrical stimulation, which mimics the mechanisms of endogenous electric field during the wound healing process through adjusting frequency, wave amplitude, lasting time or pulse type.^[^ ^46^, ^47^, ^48^ ^]^ The difference between endogenous and exogenous electric field is presented in **Table** **1**. In this section, we will introduce and discuss their mechanisms on wound healing.

**Table 1:** Comparison of endogenous and exogenous electric field.

Electrical stimulation modes	Stimulating manners	Working characteristics	Reference
Endogenous electric field	Ion transport	Electric potential difference	^[^ ^43^ ^]^
Exogenous electric field	Direct current (DC)/pulsed current (PC)/alternating current (AS)	Continuous flow of electric charge in a monophasic waveform/ Intermittent flow of charged particles with gaps in current flow/Two symmetrical electrical pulses alternating one after another to form a two‐phase waveform	^[^ ^68^, ^69^, ^70^ ^]^

### 2.1 Endogenous Electric Field

**Figure 2:**
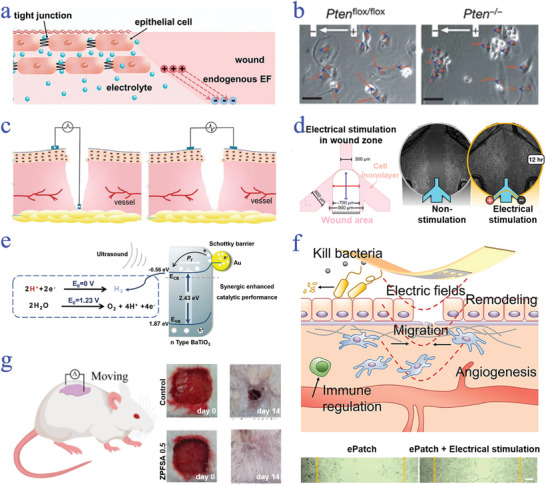
Electrical stimulation on wound healing. a) Illustration of the generation of endogenous electric field in wounds.^[^ ^50^ ^]^ Reproduced with permission: Copyright 2023, John Wiley and Sons. b) Cell migration induced by electric field regulated by PI(3)Kγ and the loss of *Pten*.^[^ ^53^ ^]^ Reproduced with permission: Copyright 2006, Springer Nature. c) Schematic diagram of two kinds of applied electrode locations on the wounds.^[^ ^67^ ^]^ Reproduced with permission: Copyright 2021, John Wiley and Sons. d) Illustration of bioelectronic microfluidic applied on the wound and the influence of DC electrical stimulation on the wound closure of keratinocytes.^[^ ^71^ ^]^ Reproduced under terms of the CC‐BY license: Copyright 2023, Royal Society of Chemistry. e) Scheme of electrocatalysis reaction derived from piezoelectric materials.^[^ ^74^ ^]^ Reproduced with permission: Copyright 2024, John Wiley and Sons. f) Schematic diagram of ePatch applied for wound healing with the cell migration under an electric field and optical images of fibroblast cells cultured with ePatch treated with/without electrical stimulation.^[^ ^75^ ^]^ Reproduced with permission: Copyright 2022, Elsevier. g) Illustration of electrical stimulation exerted on rat's skin and photographs of wound healing treated with a control group and piezoelectric dressing group.^[^ ^82^ ^]^ Reproduced with permission: Copyright 2022, American Chemical Society.

Endogenous electric field, which is abundant across tissues and cells in creatures, was identified by German physiologist Emil Du‐Bois Reymond.^[^ ^49^ ^]^ The electric current produced at wound sites can be attributed to the short‐circuits of a trans‐epidermal potential (TEP). Specifically, TEP is formed based on the asymmetric distribution of ion channels in the epithelial layer and directional ion transport (sodium, potassium, and chloride ions) through polarized epithelial cells. Once skin injury occurs, various external signals will be generated with the loss of electrolytes, resulting in the short‐circuits of TEP (**Figure** **2**a). Notably, it was demonstrated that the wound center determined the flow direction of positive charges.^[^ ^50^ ^]^ And compared with healthy skin, wound skin shows a negative potential. This means the appearance of a potential gradient triggers the flow of current from the normal skin at a certain distance away from the wound, toward the wound, thereby promoting the formation of a lateral endogenous electric field. Adjusting ion transport has proved to be useful in affecting the recovery of wound.^[^ ^51^ ^]^ Thus, endogenous ionic currents are extremely important guidance cues in wound healing.

Evidence suggests that the manipulation of endogenous electric fields has a huge impact on wound therapy.^[^ ^52^ ^]^ Research indicated that the presence of endogenous electric field could affect cell migration through the activation of Src and inositol‐phospholipid signaling (Figure 2b).^[^ ^53^ ^]^ Such cell migration induced by electric field was dependent on unignored genes including PI(3)Kγ, and the gene named phosphatase and tensin homolog (*Pten*). In addition, the inflammatory phase is accelerated through the migration of macrophages, lymphocytes, and neutrophils to wound area, shortening the process of wound healing.^[^ ^54^ ^]^ Notably, if the wounds suffer from the loss of electrolytes, resulting in the decrease of endogenous electric fields, the wounds have difficulty in healing. For instance, diabetes wounds were always hard‐healing due to their lower electrical TEP.^[^ ^55^, ^56^, ^57^, ^58^ ^]^ Collectively, researchers have concluded that endogenous electric fields play an essential role in wound healing^[^ ^59^ ^]^ and the healing mechanisms of endogenous electric fields applied on wounds are mainly due to the promotion of collagen deposition, effective cell migration and antibacterial promotion.

### 2.2 Exogenous Electric Field

Inspired by the endogenous electric field, great efforts have been dedicated to the research of exogenous electrical stimulation.^[^ ^60^, ^61^, ^62^ ^]^ Generally, the working mechanism of an exogenous electric field is to take advantage of external electric stimulation to mimic and enhance wound potential. According to the principal, various electrical stimulation devices have been developed and widely applied in wound healing and tissue engineering.^[^ ^63^, ^64^, ^65^, ^66^ ^]^ On the basis of the different locations where electrodes act on the wound, they can be divided into two categories (Figure 2c).^[^ ^67^ ^]^ Specifically, one is that the anode is placed on the healthy skin nearby the wound accompanying with the cathode to the center of the wound, which is recognized as the unidirectional current or voltage; while the other is that two electrodes are all placed on healthy skins on both sides of the wounds, which is characterized as bidirectional current or voltage. Commonly, the electrical stimulation derived from such two categories has a similar impact on wound healing.

In addition, these developed devices working mainly rely on external power sources, which can be classified as three electrical stimulation working modes, including DC, PC, and AS.^[^ ^68^, ^69^, ^70^ ^]^ Among them, DC and PC are commonly used electrical energy‐providing manners for electrical stimulation. DC has been demonstrated as an excellent candidate with immense potential due to its capability on guiding directional migration of epithelial cells. Recently, Asplund's group proposed a bioelectronic microfluidic platform based on direct current stimulation.^[^ ^71^ ^]^ Results showed that the group with DC electrical stimulation greatly enhanced the wound healing rate of diabetes rats nearly triple times compared with the group without intervention (Figure 2d). This indicated that DC electrical stimulation was a viable pathway to accelerate wound healing. Besides, Chen et al. adopted a drug‐loaded bacterial cellulose‐based bandage for promoting wound healing by DC stimulation (600 µA) to force the effective release of growth factors.^[^ ^72^ ^]^ It was illustrated that the intensity of DC within 200–800 µA was safety and effective for wound treatment. Despite these advancements, the application of DC electrical stimulation is limited because the sustained charge buildup will generate a continuous thermal effect around wounds, which has a negative effect on skins. Different from DC with unidirectional current, PC is a kind of bidirectional current possessing high pulse voltage and low pulse current output, which has little side effect and less invasiveness of electrodes, giving a promising approach for clinical treatment in wound healing. Many works concerning PC‐based electrical stimulation for wound healing have been reported, which proved the advantages of electrotherapy for skin treatment.^[^ ^70^, ^73^ ^]^

Not only endogenous electric field has a significant role in wound healing, but also exogenous electric field can also induce a series of effects in wound healing phases, thus improving wound healing process. For instance, effective electrical stimulation can downregulate inflammation through scavenging reactive oxygen species (ROS) and secretion of inflammation factors. Shi et al. reported that by taking advantage of piezoelectric materials to generate electric field, the produced hydrogen from electrocatalysis was capable of reducing ROS, further regulating inflammatory microenvironment (Figure 2e).^[^ ^74^ ^]^ Massive bacteria can disturb the wound environment and electrical stimulation has been proven to be a useful means to kill them, achieving ideal wound treatment. Especially, the antibacterial effect is associated with the durable time and density of electrical stimulation. Even, the most well‐known influence of electrical stimulation on wound healing involves the guidance of biological cues for cells, enhancing cell migration and proliferation. Like, group of Khademhosseini^[^ ^75^ ^]^ developed a flexible patch (ePatch) composed of silver nanowires to achieve electrical stimulation (Figure 2f). A higher migration rate of fibroblast cells was observed and most cells grew in a directed manner under electrical stimulation. Meanwhile, they illustrated that pulsed electrical stimulation showed a positive impact on cell proliferation via affecting cell signaling pathways, contributing to a rapid would closure. Generally, apart from fibroblast cells, such electrical signal responsiveness is related to some other cells, including epithelial and endothelial cells.^[^ ^76^ ^]^ In addition to cell signaling pathways, gene signaling pathways can be activated by electrical stimulation. Poulas et al. demonstrated that microcurrent stimulation contributed to the upregulation of genes concerning transforming growth factor‐βand Hedgehog signaling pathways, thus promoting wound healing.^[^ ^77^ ^]^ Zhang group also proved that electrical stimulation can modulate multiple gene expressions across the wound healing process.^[^ ^78^ ^]^ Furthermore, electrical stimulation is also helpful for angiogenesis via improving the expression of vascular endothelial growth factor (VEGF).^[^ ^79^ ^]^

To the best of our knowledge, the scar formation is highly associated with mass collagen deposition and disorderly remodeling of extracellular matrix (ECM) during wound remodeling stage.^[^ ^80^, ^81^ ^]^ Electrical stimulation has been demonstrated with the capability of inducing regular collagen deposition and improving the expression of growth factors, which are beneficial for scar elimination. Fan et al. presented a piezoelectric wound dressing called ZPFSA for preventing scar formation through the action of microcurrent generated from dual piezoelectric release on rat's wounds (Figure 2g).^[^ ^82^ ^]^ The after‐treated wound showed rapid scarless wound closure due to bioelectric stimulation derived from the piezoelectric response, which helped the prevention of scar tissue. In addition, electrical stimulation is also a powerful tool for achieving expected drug release by loading drugs into electric‐responsive carriers, thus promoting wound healing. Many drug release systems based on electrical stimulation for realizing the ideal wound treatment effect have been proposed.^[^ ^83^, ^84^, ^85^ ^]^

## 3 Types of Electrical Microneedles

Compared with transcutaneous electrical stimulation, invasive electrical stimulation has been reported with higher electric field density and parallel distribution, thus contributing to better wound therapeutic effects.^[^ ^86^ ^]^ Microneedles, as a kind of attractive transdermal drug delivery device with painless and effective invasiveness, have been widely applied in various skin disease therapy.^[^ ^87^, ^88^, ^89^ ^]^ Given the ability to create transdermal pathways, attention is concentrated on the integration of microneedles with electric field to build electrical microneedles. A series of electrical microneedles have been put forward, which can be mainly classified into conductive‐driven, triboelectric‐driven, piezoelectric‐driven microneedles with respect to their working principles. In this section, we will provide a detailed introduction and comparison of their differences.

### 3.1 Conductive‐Driven Microneedles

Conductive‐driven microneedles are composed of microneedles with electrically conductive fillers, such as conductive polymers, metals, carbon, MXene, etc. Due to flexible adjustability in terms of the conductive fillers, dopants, and cross‐linking state, the performance of microneedles can be easily controlled, involving physical and chemical properties, electrical effects, and biological functions, for meeting various applications. We will present a brief introduction of these conductive‐driven microneedles.

#### 3.1.1 Conductive Polymer‐Based Conductive Microneedles

**Figure 3:**
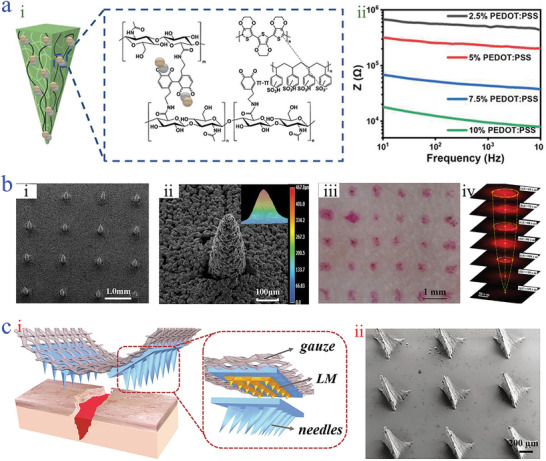
Conductive‐driven microneedles based on conductive polymers and metals. a) Scheme of conductive dopamine hyaluronic acid hydrogel microneedles embedded with PEDOT:PSS i) and electrochemical impedance spectroscopy measurement of the conductive hydrogel microneedles with the concentration change of the PEDOT:PSS ii).^[^ ^98^ ^]^ Reproduced with permission: Copyright 2022, John Wiley and Sons. b) Scanning electron microscope (SEM) image i) and enlarged image ii) of the porous Ti microneedles; the optical image of rabbit skin after punctured by porous Ti microneedle iii); the fluorescence photo of drug release of Ti microneedle at different depth iv).^[^ ^105^ ^]^ Reproduced under terms of the CC‐BY license: Copyright 2017, Public Library of Science. c) Illustration of LM‐based PEGDA microneedles with tilted triangle pyramid tips i); SEM image of the LM‐based PEGDA microneedle ii).^[^ ^110^ ^]^ Reproduced with permission: Copyright 2021, Elsevier.

Conductive polymers are a type of organic material with a delocalized electronic structure that enables electrons to stay and transfer in their main chains.^[^ ^90^, ^91^ ^]^ Generally, several kinds of conductive polymers have roused increasing attention such as polyaniline (PANI), polypyrrole (PPy), and poly(3,4‐ethylenedioxythiophene) (PEDOT), with their outstanding features including tunable electrical properties, good biocompatibility, and solution processability.^[^ ^92^, ^93^, ^94^ ^]^ The combination of these conductive polymers with hydrogels allows for the construction of continuous 3D interconnected conductive networks. Especially, taking advantage of diverse engineering methods like template molding, 3D printing, microfluidics, such conductive polymers‐based hydrogels can be manufactured into desirable micro/nanostructured materials, meeting the needs of practical applications.^[^ ^95^, ^96^, ^97^ ^]^ Thus, multiple conductive polymer‐based conductive microneedle systems have emerged and shown outstanding electrical behaviors. In a study by Poudineh et al.,^[^ ^98^ ^]^ a conductive hydrogel microneedle adopted PEDOT: polystyrene sulfonate (PSS) as the water‐dispersible conductive polymer was fabricated through the template molding (**Figure** **3**a‐i). Owing to the presence of PEDOT:PSS, the resultant microneedle exhibited enhanced conductivity (Figure 3a‐ii), possessing the potential for acting as the working electrode of sensor. Notably, the conductivity and mechanical strength of microneedles were influenced by the dispersion of conductive polymers in hydrogels, which determined the successful connection of conductive networks and the capability of bearing external stresses. In addition, using a chemical deposition or solution coating approach, conductive polymers can be integrated into microneedles. Leese et al. endowed in situ chemical polymerization and drop casting methods to impart 3D‐printing‐derived microneedles with PPy and PEDOT:PSS polymers coating, thus endowing the microneedle with conductivity.^[^ ^99^ ^]^ The coated surface of microneedles showed a decreased roughness and quite uniform morphology, which was associated with their hydrophilicity and conductivity.

#### 3.1.2 Metals‐Based Conductive Microneedles

Due to their intrinsic excellent conductivity, malleability, superior mechanical performance, and prominent optical and thermal properties,^[^ ^100^, ^101^, ^102^ ^]^ metals and metal alloys have exhibited promising possibilities in the design of various conductive microneedles. The metals and metal alloys employed for microneedles mainly involve stainless steel, titanium, tantalum, nickel, as well as liquid metals (LMs). Previous research reveals that stainless steel is one of the most commonly used metals for conductive microneedles with the advantages of commercially available and robust puncture ability. For example, Zhang et al. fabricated a hollow stainless‐steel microneedle, which exhibited great insertion capability without dependence on external forces and remained undamaged state.^[^ ^103^ ^]^ Similarly, porous metallic microneedles containing medical‐grade stainless steel were presented by O'Cearbhaill.^[^ ^104^ ^]^ Additionally, researchers adopted a micro‐molding method to fabricate a porous titanium (Ti) microneedle array with tips’ height of about 496.6 µm, whose surface was observed with a relatively rough morphology (Figure 3b‐i,b‐ii).^[^ ^105^ ^]^ The Ti microneedle could penetrate the rabbit skin, which was beneficial for drug release in subcutaneous depth (Figure 3b‐iii,b‐iv). Comparatively, LMs are different from these metal materials, which are a kind of metal alloy having the impressive feature of flowability with a melting point at normal atmospheric temperature. Supported by the properties of high conductivity, shape‐deformability, and low toxicity, LMs have been famous in wide applications such as soft robots, electronic skins, and flexible medical devices.^[^ ^106^, ^107^, ^108^, ^109^ ^]^ Given these, our group developed LMs‐based conductive poly(ethylene glycol) diacrylate (PEGDA) microneedles by using a top‐down multiple‐mold‐guided photolithography approach (Figure 3c‐i).^[^ ^110^ ^]^ The acquired microneedles were distributed with W‐shaped LM rails and the microneedle tips displayed tilted triangle pyramid structures (Figure 3c‐ii). Such distinctive architecture was proved with satisfactory interconnected conductive pathways and enhanced skin penetration behavior, which facilitated further electrical stimulation and fixation on in vivo skin wounds.

#### 3.1.3 Carbon‐Based Conductive Microneedles

**Figure 4:**
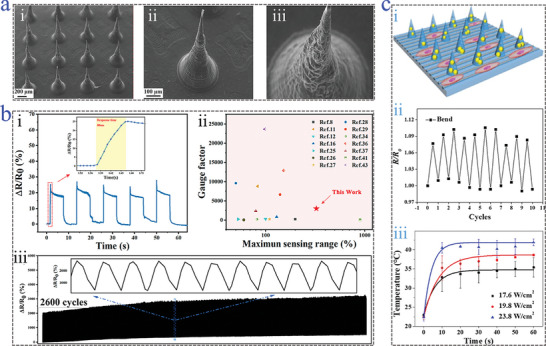
Conductive‐driven microneedles based on carbon materials. a) SEM images of GO‐HAMA microneedle array i); enlarged image ii); tip's surface iii).^[^ ^115^ ^]^ Reproduced with permission: Copyright 2023, Elsevier. b) Relative resistance changes with the response time of TPU microneedles with CNTs i); comparison of gauge factor in previous works and TPU‐CNTs microneedles ii); relative resistance variations when experiencing 2600 cycles of stretching and releasing iii).^[^ ^116^ ^]^ Reproduced with permission: Copyright 2022, American Chemical Society. c) Illustration of HAMA‐CNTs microneedles cultured with cells i); relative resistance changes of HAMA‐CNTs microneedles suffering from bending ii); light‐to‐thermal conversion curve of HAMA‐CNTs microneedles under light irradiation with different intensities iii).^[^ ^117^ ^]^ Reproduced with permission: Copyright 2023, Elsevier.

Carbon nanotubes (CNTs) and graphene are believed as appealing 1D and 2D carbon‐based materials in light of their remarkable electrical property, good flexibility, and ideal processability, greatly contributing to the advancement of the research field of flexible sensors.^[^ ^111^, ^112^, ^113^ ^]^ Typically, well‐known approaches for the preparation of carbon‐based conductive microneedles involve spray coating and solution mixing. Fang and colleagues employed CNTs as conductive fillers into chitosan solution to form a bio‐interface on the surface of microneedles.^[^ ^114^ ^]^ Similarly, Lei et al. fabricated a hyaluronic acid methacrylate (HAMA) microneedle encapsulated with graphene oxide (GO).^[^ ^115^ ^]^ As we can see from **Figure** **4**a, the microneedle tips were covered by GO, exhibiting a rough and porous surface. In addition, to bridge the conductive paths, CNTs were incorporated into thermoplastic polyurethane (TPU) microneedles, which greatly enhanced their conductive sensitivity (gauge factor_max_ of 2911) and improve their mechanical durability (bearing 2600 stretching cycles under 50% strain) (Figure 4b).^[^ ^116^ ^]^ It is especially advantageous that CNTs can be arranged in an orderly manner, which is conducive to promoting cell or tissue growth. Such unique feature was verified by our group that aligned CNTs sheet served as the HAMA microneedles’ basement, forming the resultant conductive microneedle patches.^[^ ^117^ ^]^ Ascribed to the performance of aligned CNTs, the microneedle patches were imparted with great conductivity and photo‐thermal responsiveness, as well as the capability of guiding he orientation growth of fibroblasts (Figure 4c).

#### 3.1.4 MXene‐Based Conductive Microneedles

**Figure 5:**
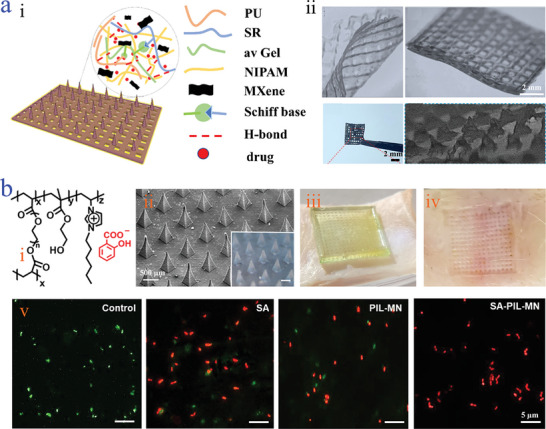
Conductive‐driven microneedles based on MXene and electrolyte/polyelectrolyte. a) Scheme of the MXene‐integrated PU/ avGel microneedle i); SEM images of the MXene‐integrated PU/ avGel microneedle ii).^[^ ^120^ ^]^ Reproduced with permission: Copyright 2022, American Chemical Society. b) Chemical structure of the SA‐PIL microneedle patch i); SEM image of the SA‐PIL microneedle patch ii); the optical images of SA‐PIL microneedle patch punctured in rabbit skin iii) and after the removal of SA‐PIL microneedle patch iv); the bacterial fluorescence images of *four groups against P. acnes* v).^[^ ^122^ ^]^ Reproduced with permission: Copyright 2020, Elsevier.

Benefitting from the excellent metallic conductivity, large active surface area, high photo‐thermal transition efficiency, and good water dispersibility, MXene, a family of 2D transition metal carbonitrides, has been regarded as a favorable electrode material for the design of conductive microneedles.^[^ ^118^, ^119^ ^]^ For instance, He's team mixed the MXene solution into the pregel solution containing water‐soluble polyurethane (PU) and aloe vera gel (avGel) (**Figure** **5**a‐i).^[^ ^120^ ^]^ With the help of 3D printing technology, a MXene‐integrated microneedle with composite structures showing interconnected microporous scaffold basement and microneedle tips was obtained (Figure 5a‐ii). The special gel compositions and the introduction of MXene contributed to the multiple functions of the acquired microneedles, such as light‐responsiveness, self‐healing, drug‐carrying ability as well as electrical property. It was worth mentioning that photo‐thermal conversion capacity enhanced the value of MXene‐integrated microneedles, making them tremendous potential in acting as intelligent drug delivery materials. There has emerged much research on exploring the unique property of MXene microneedles, and great progresses has been achieved, further demonstrating their superior potential.

#### 3.1.5 Electrolyte/Polyelectrolyte‐Based Conductive Microneedles

Electrolyte/polyelectrolyte hydrogels are prominent components of microneedles, in which fast and massive ion migration occurs in their channel networks. Such ion migration results in the generation and transmission of electrical signals, likely attributed to the closely interactions of polyelectrolytes with water molecules. In addition to ionic conductivity, extra appealing features have been explored, including highly tailorable physiochemical properties, salient flexibility, and outstanding stretchability.^[^ ^121^ ^]^ These attractive merits empower various electrolyte/polyelectrolytes to construct conductive microneedles. Yan et al. proposed a poly(ionic liquid) (PIL)‐based microneedles by the photo‐crosslinking method.^[^ ^122^ ^]^ PIL, a class of polymer consisting of anions and cations, not only shows high ionic electronic performance, but also has antimicrobial capability derived from the electrostatic interactions between PIL and bacterial cell walls, further destroying the bacteria.^[^ ^123^ ^]^ The PIL in microneedles used was 3‐heptyl‐1‐vinylimidazolium bromide (Im‐C6), which was an imidazolium type ionic liquid monomer (Figure 5bi). Thanks to these, the obtained conductive SA‐PIL microneedles exhibited adjustable mechanical ability that allowed to penetrate skin without damage and efficient antibacterial property against *Propionibacterium acnes (P. acnes)* (Figure 5bii‐v). Besides, the freezing resistance of SA‐PIL microneedles could be dramatically improved based on the presence of ions, which would be beneficial for them to accommodate the complex and extreme environment. More attractively, different from electronic conductive microneedles featured with dark and hydrophobic compositions, ionic conductive microneedles exhibited good transparency and hydrophilicity, highly broadening their application areas.

### 3.2 Triboelectric‐Driven Microneedles

Triboelectric is a kind of natural phenomenon, which can convert mechanical behaviors to electrical signals without external devices providing power sources.^[^ ^124^ ^]^ Such distinctive feature is originated from the synergistic influence of contact electrification and electrostatic induction. Profiting from the principle and their properties such as self‐powered ability, low cost, portability, implantable and mild electrical stimulating condition, various triboelectric‐driven devices have been proposed and triboelectric nanogenerators (TENG) are one of the most commonly used energy collection technology.^[^ ^125^, ^126^ ^]^ Among them, triboelectric‐driven microneedles are increasingly prevalent due to the more convenient usage of electrical stimulation into deeper tissue through microneedle tips’ penetration, which shows superior advantages over many traditional electrical therapy materials. Notably, different from the above‐mentioned conductive‐based microneedles that commonly need battery‐powered devices, thus posing potential cellular and physiological risks, triboelectric‐driven microneedles present self‐powering electrical energy, which are more suitable personalized healthcare, implantable and surface bioelectronics. According to their working mechanisms, four fundamental modes of triboelectric‐driven microneedles have been widely explored, including vertical contact‐separation mode, lateral sliding mode, single electrode mode, and free‐standing mode. Given these, the working mechanisms of triboelectricity are the prerequisite for the deeply understanding.

#### 3.2.1 Working Mechanism of Triboelectricity

Commonly, the appearance of triboelectric phenomenon is originated from contact between most materials, such as silk, plastic, metals, wood, etc.^[^ ^127^ ^]^ Accordingly, electron transferring from one material to another is recognized as the main cause of triboelectricity. It can be anticipated that surface charge densities of the triboelectric behaviors are influenced by the charge difference between these materials, which possess positive and negative charges. Taking inspiration from the triboelectric behaviors, TENG emerges and achieves greatly advancement, which are typically created by triboelectrically charged surface and electrodes. The generation of TENG electric output is dependent on the changes of electrostatic potentials caused by the movement on charged TENG surface. Theoretically, the design of TENG relies on the Maxwell displacement current, as illustrated in Equation ((1), (2), (3), (4)):^[^ ^128^ ^]^

(1) $${\mathrm{Gauss}}^{\prime }s\;\mathrm{law}\;▿\times D=\rho_{f}$$

(2) $${\mathrm{Gauss}}^{\prime }s\;\mathrm{law}\;\mathrm{correlated}\;\mathrm{to}\;\mathrm{magnetism}\;▿\times B=0$$

(3) $${\mathrm{Faraday}}^{\prime }s\;\mathrm{law}\;▿\times E=-\frac{\partial \;B}{\partial \;t}$$

(4) $$\mathrm{Ampere}\;\mathrm{Maxwell}\;\mathrm{law}\;▿\times H=J_{f}+\frac{\partial \;D}{\partial \;t}$$

where ▿ represents vector differential operator, *D* accounts for the potential shift vector, ρ_*f*_ is the free electric charge density, the magnetic field is denoted as *B*, E is the electric field, H is the magnetizing field and *J_f_* describes the free electric current density. The above equations are time‐independent and operate in the condition of stationary media of electromagnetic waves. In the case of variation time and considering electrostatic charges derived from contact electrification, an essential parameter is pointed out by Wang's group.^[^ ^129^ ^]^ That is *P_s_*, which is characterized as mechano‐induced polarization of the medium. Based on this, Maxwell equation is updated and the modified Maxwell's equation promotes the formation of the fundamental theory of TENG, which is described below:

(5) $$\nabla \cdot D\;=\rho_{f}-\nabla \times P_{s}$$

(6) ▿×B=0

(7) $$▿\times E=-\left(\frac{\partial \;}{\partial \;t}+\nu \times ▿\right)B$$

(8) $$▿\times H=J_{f}+\left(\frac{\partial \;}{\partial \;t}+\nu \times ▿\right)\left(P_{s}+D\right)$$

where ν is defined as movement velocity of the medium.

According to the modified Maxwell's equation, diverse TENG dedicated to turning mechanical energy into electrical energy have been proposed and achieved enormously attributes in numerous applications.^[^ ^130^, ^131^, ^132^ ^]^ Particularly, TENG is a promising choice for developing microneedles with electricity. Specifically, triboelectrification is a critical aspect for the design of TENG‐driven microneedles, whose working modes are dependent on structural design and mechanical energy source. In general, there have been four working modes involving vertical contact‐separation mode, lateral sliding mode, single electrode mode, and free‐standing mode. Therefore, we will introduce recent advances of these triboelectric‐driven microneedles based on these four working fundamental modes.

#### 3.2.2 Vertical Contact‐Separation Mode‐Based Microneedles

**Figure 6:**
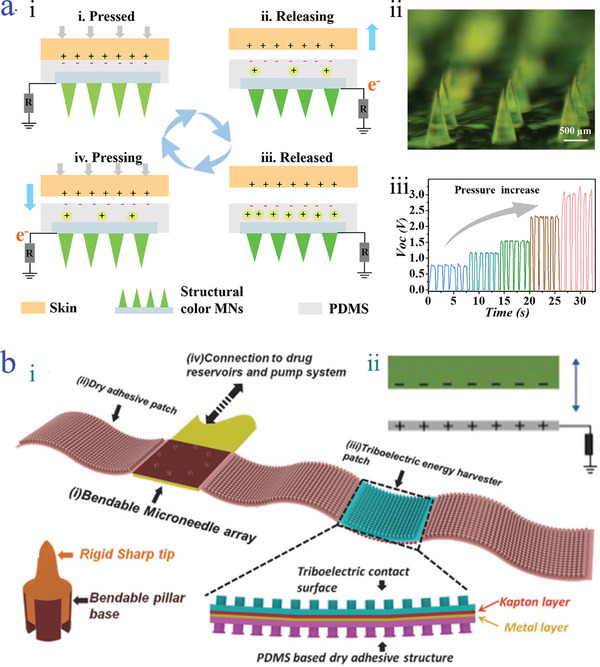
Triboelectric‐driven microneedles. a) Working mechanism of vertical contact‐separation mode‐based microneedles i); the optical image of PAM‐PEGDA‐LiCl microneedle ii); open‐circuit voltage (*Voc*) of PAM‐PEGDA‐LiCl microneedle under the pressure iii).^[^ ^134^ ^]^ Reproduced with permission: Copyright 2023, Elsevier. b) The schematic diagram of the self‐powered PDMS microneedle wearable device i); the working principle of the self‐powered PDMS microneedle wearable device with single‐electrode mode ii).^[^ ^137^ ^]^ Reproduced with permission: Copyright 2016, John Wiley and Sons.

Vertical contact‐separation mode is one of the most universal contact‐induced triboelectrification.^[^ ^133^ ^]^ In regard of structural design, two kinds of dielectric materials coating with metal electrodes are needed and they must be placed on both vertical sides. Electron transfer mainly accounts for the surface charging mechanisms (**Figure** **6**a‐i).^[^ ^134^ ^]^ In detail, when the contact behavior occurs, opposite surface charges on the two triboelectric surfaces are generated. Once separation begins with the external force, an induced potential difference between two electrodes gradually appears. At this moment, to balance the electrostatic field, the electrons are forced to flow from one electrode to another, forming a reverse potential difference. In case re‐contact happens, such potential difference will be disappeared and backflow of electrons undergo. Much evidence has illustrated that by taking advantage of the contact‐separation behavior accompanied by electron transfer, triboelectric effect emerges and an electrical signal can be detected.

A typical example of vertical contact‐separation mode‐based microneedles was presented by our group (Figure 6a‐i).^[^ ^134^ ^]^ The microneedle was composed of a double‐layer composite structure consisting of the polyacrylamide‐PEGDA‐lithium chloride (PAM‐PEGDA‐LiCl) as the main body layer, and polydimethylsiloxane (PDMS) as the external protective layer (Figure 6a‐ii). Due to the well‐known strongly negative triboelectric property of PDMS, the proposed microneedle showed distinct and stable electron transmission through vertical contact‐separation mechanical behavior between the PDMS layer and finger's skin (Figure 6a‐iii). It was noteworthy that the triboelectric effect of these microneedles could contribute to controllable and effective drug delivery benefiting from the electrostatic repulsion between drugs and unshielded residual negative charges of PDMS. Given this merit, the triboelectric microneedle was beneficial for the treatment of diseases. Similarly, another bilayer triboelectric microneedle was obtained by employing stainless‐steel microneedle as the substrate and solvable polyvinylpyrrolidone (PVP) as the overlying layer.^[^ ^135^ ^]^ The generation of triboelectric signals was derived from the introduction of polytetrafluoroethylene‐polymethyl (PTFE‐PET), which is a kind of negatively charged material.

#### 3.2.3 Single‐Electrode Mode‐Based Microneedles

Single‐electrode mode is a relatively simple and practical operating mode, whose triboelectric layers rely on dielectric/conductor and dielectric/dielectric.^[^ ^136^ ^]^ The unique advantage of single‐electrode mode over others involves the simplicity of structural design, which adopts the ground as a reference electrode without an external circuit. The potential difference is originated from the flow of electrons between the electrode and the ground (Figure 6b‐ii).^[^ ^137^ ^]^ Evidence suggests that the triboelectricity is influenced by surface structures and materials. Microneedles compared with flat surfaces have a limited contact area with a certain surface and show easy deformation ability, beneficial for enhancing triboelectric properties. For instance, Lee et al. proposed a self‐powered wearable microneedle device, mainly composed of a bendable microneedle patch with a soft PDMS four‐beam pillar substrate and rigid SU‐8 tips and triboelectric energy harvester (Figure 6b‐i).^[^ ^137^ ^]^ The structural design of the microneedle patch enabled effective deformation and skin penetration. To achieve the single‐electrode mode and establish triboelectric energy collector, a copper (Cu) layer was attached on the PDMS layer. Through contacting and pressing with a finger, an electrical signal was generated by charge transport. In detail, upon contacting with skin, electrons from PDMS were transferred to the skin, which induced the variations of negative charges on PDMS, further driving the free electrodes on the Cu electrode to flow to the ground, thus detecting output power.

Although single‐electrode mode has great applicability, the output performance of microneedle in this mode is restricted. To solve the problem, researchers have concentrated on the adjustment of microneedle tip morphologies to obtain a greater contact area, thereby improving the triboelectric capability. Cho's group tuned the surface geometries of microneedle tips to micro‐dominoes and employing the single‐electrode mode, which greatly enhanced triboelectricity.^[^ ^138^ ^]^ Compared with flat surface with small roughness, domino‐based TENG microneedle exhibited an exceedingly high output power, which could be ascribed to the enlargement of the contact area when suffering from deformation. Given this, the output performance could be controlled by adjusting the surface morphology.

#### 3.2.4 Lateral Sliding Mode‐Based Microneedles

**Figure 7:**
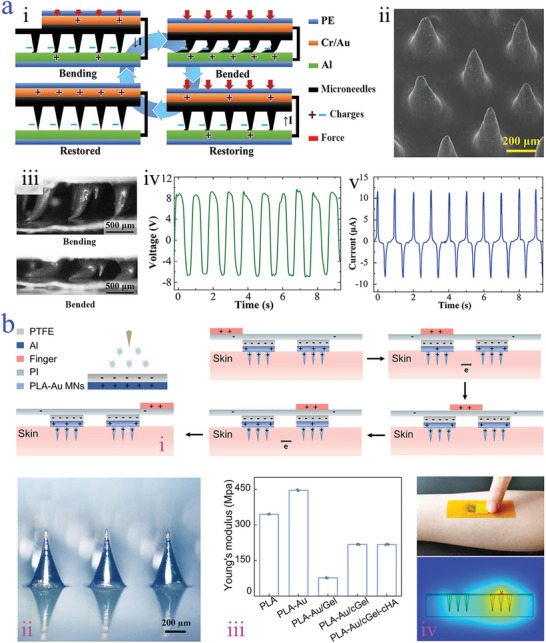
Triboelectric‐driven microneedles. a) Working mechanism of lateral sliding mode‐based microneedles i); SEM images of the PDMS‐CMRF microneedle array ii) and of the PDMS‐CMRF microneedle array experiencing bending before and after iii); the *Voc* curve of the PDMS‐CMRF microneedle array iv); the short‐circuit current curve of the PDMS‐CMRF microneedle array v).^[^ ^139^ ^]^ Reproduced with permission: Copyright 2019, John Wiley and Sons. b) Schematic diagram of the free‐standing mode working mechanism of sf‐TENG i); optical image of PLA‐Au‐based microneedle ii); Young's modulus of microneedles iii); photos and COMSOL simulation image of the working process of sf‐TENG with the movement of the finger iv).^[^ ^142^ ^]^ Reproduced under terms of the CC‐BY license: Copyright 2022, Springer Nature.

A similar structural design of contact‐separation mode is observed from lateral sliding mode and the difference between them is the direction of applied force. Specifically, the direction of the applied force of contact‐separation mode is vertical to triboelectric layer, while a planar movement of lateral sliding mode performs between the surfaces (**Figure** **7**a‐i).^[^ ^139^ ^]^ Notably, Jiang and colleagues found that the output power of the lateral sliding mode was higher than that of contact‐separation mode, which may be ascribed to more charge generation during the sliding operation process. They proposed a bendable PDMS microneedle array, which was a key component of triboelectric device. In particular, the PDMS microneedle array was assembled by magnetization induction of curable magnetorheological fluid (CMRF) and it was characterized that an orderly arrangement morphology with cones and roughness surfaces was observed (Figure 7a‐ii).^[^ ^139^ ^]^ Further, 7 microneedles per square millimeter with an average height of about 900 µm illustrated the highest electrical output performance. To fabricate microneedle‐based triboelectric device, the polyethylene (PE) film was selected as the triboelectric layer with the aluminum (Al) film as the bottom counter‐electrode, while Cr/Au layer was coated on the substrate of microneedle as the top electrode (Figure 7a‐i). Due to the proved excellent mechanical properties in terms of elasticity and bendability, the acquired microneedle‐based triboelectric device could stably withstand external forces and microneedle tips exhibited a bendable‐sliding state (Figure 7a‐iii). Such mechanical deformation behavior was beneficial for the enlargement of the friction, contributing to higher output current density (Figure 7a‐iv, a‐v). Similar magnetization‐induced self‐assembled microneedles were presented by Wong et al.,^[^ ^140^ ^]^ the difference of which was that the size of per microneedle was larger than the previous research. They fabricated a high‐aspect‐ratio microneedle array with 1.2 mm length. When experiencing the pressing forces, the microneedle‐based TENG performed bending‐sliding‐friction behavior, demonstrating improved electrical generation capability resulting from the scale‐up of specific surface area.

#### 3.2.5 Free‐Standing Mode‐Based Microneedles

Free‐standing mode shows a huge difference compared with others, which is a natural friction consisting of an independent moving triboelectric layer and two stationary tribo‐electrodes.^[^ ^141^ ^]^ Two configurations can be classified according to the moving directions of the independent moving triboelectric layer, including lateral sliding and vertical contact‐separation types. Benefitting from the distinctive structures, the power source of free‐standing mode originated from natural friction instead of external mechanical‐driven electrification, which shows superior advantages in terms of reduced surface wear, long‐term stability, and great electrical energy collection. Recent research reported by Wang et al. has illustrated sliding free‐standing mode‐based microneedles with double‐layer structure, which are composed of the polylactic acid‐gold (PLA‐Au) microneedle covered with drug‐loaded microneedles consisting of gelatin cross‐linked HA (sf‐TENG) (Figure 7b‐i).^[^ ^142^ ^]^ PTFE was used as the dielectric layer, while the triboelectric layers were the finger and polyimide film (PI). When the sliding movement was applied by the finger, the potential difference occurred, producing an output electric field (Figure 7b‐iv). The attractive feature was that the microneedle tips showed ideal mechanical strength with Young's modulus of 100 MPa (Figure 7b‐iii), which was sufficient to puncture into the dermis with low resistance about 10 kΩ. Such feature contributed to effective transcutaneous electrical stimulation.

Given these studies of four fundamental modes, triboelectric‐driven microneedles have achieved extensive advancements, which will be potential in a wide range of applications. However, there is still a disparity between the research stage and practical use. For example, triboelectric‐driven microneedles depend on external energy and mechanical forces to acquire triboelectric effect, which brings low energy convention efficiency. Besides, material and structural durability are urgently needed to extend the lifespan and improve the reliability of the triboelectric‐driven microneedles.

### 3.3 Piezoelectric‐Driven Microneedles

The piezoelectric effect was revealed by Curie brothers in 1880,^[^ ^143^ ^]^ which is capable of transforming mechanical energy into electricity by taking advantage of piezoelectric materials. Specially, the electric microenvironment can be set up for a broad of applications, such as tissue repair and remineralization, bone regeneration, and cellular restoration. These piezoelectric materials range from inorganic materials (zinc oxide (ZnO), piezoceramic barium titanate (BaTiO_3_), sodium niobite (NaNbO_3_), cadmium sulfide (CdS), molybdenum disulfide (MoS_2_), etc.) to organic biomaterials (silk, collagen, amino acids, etc.) and polymers (polyvinylidene fluoride (PVDF), trifluoroethylene (TrFE), hexafluoropropylene (HFP), etc.).^[^ ^144^, ^145^, ^146^ ^]^ These piezoelectric materials show outstanding piezoelectric properties owing to the electrical polarization of non‐centrosymmetric crystal. The unique electromechanical performance is conducive to the self‐powered ability supported by converting mechanical stimuli into electrical polarization or even harvesting the body's or tissues’ movement. Thanks to these attributes, various piezoelectric devices called piezoelectric nanogenerators (PENG) have been developed, which exhibit great promise in biomedical applications, especially in wearable and wireless implantable fields.^[^ ^147^, ^148^, ^149^ ^]^ Notably, taking advantage of diverse physical stimuli, such as ultrasound and acoustic wave, an appealing potential of these devices based on piezoelectric materials is acknowledged by their activation to generate electricity, which is valuable for improving wound healing. In parallel, extensive efforts are invested in combining them with microneedles, which can further enhance therapeutic effects relying on the increase of penetration depth accompanied by drug delivery. In this section, recent studies concerning piezoelectric‐driven microneedles and the derived devices are introduced involving their constructions and working features.

**Figure 8:**
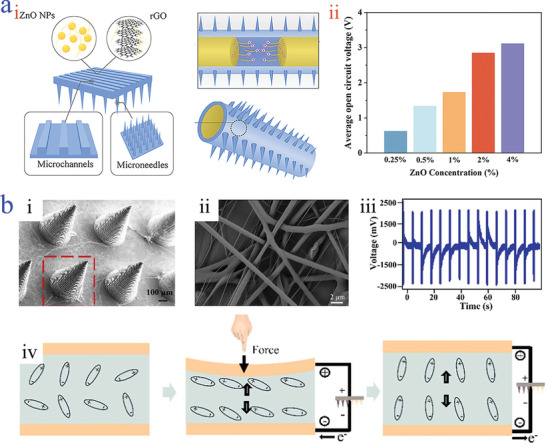
Piezoelectric‐driven microneedles. a) Illustration of the bioinspired piezoelectric‐driven polycaprolactone/ZnO microneedle i); the relationship of average *Voc* and ZnO concentration ii).^[^ ^150^ ^]^ Reproduced with permission: Copyright 2024, American Chemical Society. b) SEM images of PLA‐Au‐PPy microneedle i) and PVDF/CNT electrospun fibers ii); voltage signals responding to mechanical force iii); working mechanism of PLA‐Au‐PPy microneedle‐based PENG iv).^[^ ^151^ ^]^ Reproduced with permission: Copyright 2024, Elsevier.

Applying mechanical energy to induce electrical energy is a typical feature of piezoelectric effect. It is well‐known that piezoelectric effect can be generated by some creatures such as sea cucumbers via their muscle exercise, which inspires great attention on imitating their structures. Using the principle, Fan et al. developed a bioinspired piezoelectric‐driven microneedle, which adopted rolling microneedles with tips facing outward to mimic the morphology and function of sea cucumbers (**Figure** **8**a‐i).^[^ ^150^ ^]^ The piezoelectric effect was achieved by the introduction of ZnO nanoparticles with non‐centrosymmertic atomic structure. It was demonstrated that the bending deformation behavior contributed to producing current output stemming from the accumulation of an equal amount of opposite charges of ZnO nanoparticles. The output current and voltage performance showed a positive correlation with the concentration increase of ZnO nanoparticles (Figure 8a‐ii). Apart from bending mechanical behavior, pressure stimulation provoking the generation of electrical energy is another universal solution for piezoelectric‐driven microneedles. Zhao's group reported a self‐powered system by integrating PLA microneedles coated with Au as electrode component, which loaded with PPy as drug delivery component, and PVDF/CNT electrospun fibers as piezoelectric component (Figure 8b‐i).^[^ ^151^ ^]^ The incorporation of CNT could greatly improve the piezoelectric properties (Figure 8b‐ii,b‐iii). Once experiencing external pressure, a polarization state of PVDF dielectric occurred resulting in the movement of ion balance from their neutral positions, causing the production of the electric field. By the way, in case of removing external pressure, it restored to its original uncharged state (Figure 8b‐iv). It was illustrated that the piezoelectric voltage was proportional to the applied pressure intensity. When the finger pressing pressure was 2.942 kPa, the output open circuit voltage could reach to ≈2200 mV.

**Figure 9:**
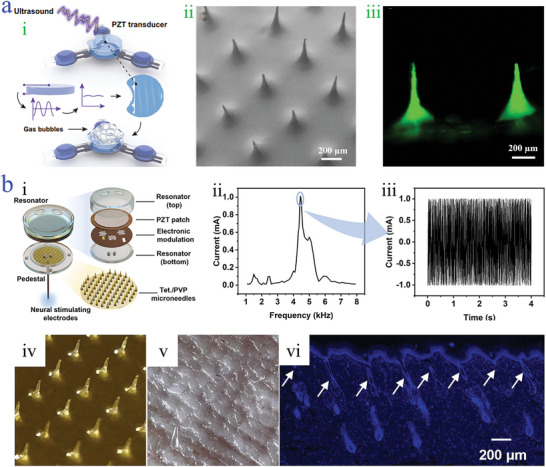
Piezoelectric‐driven microneedles. a) Scheme of the electrolysis reaction generated from ultrasonic of the riboflavin loaded PVP microneedle patch i); SEM image of the riboflavin loaded PVP microneedle array ii); fluorescence image of the PVP microneedle patch with drug loading iii).^[^ ^152^ ^]^ Reproduced under terms of the CC‐BY license: Copyright 2024, Springer Nature. b)Schematic diagram of piezoelectric tetracycline loaded PVP microneedle system i); the output current under different acoustic frequencies ranging from 1 to 8 kHz ii); the output current under the same acoustic frequency of 4450 Hz iii); optical image of the tetracycline loaded PVP microneedle array iv); photograph of the rat skin after puncturing with tetracycline loaded PVP microneedle v); the fluorescence staining of tissue in (v) vi).^[^ ^154^ ^]^ Reproduced with permission: Copyright 2023, Elsevier.

In addition to the external mechanical response, ultrasound is a fascinating means to realize effective piezoelectric effect in a wirelessly controllable and battery‐free manner. With the aid of an external ultrasound source, more smart microneedles with self‐powered ability can be created. For instance, a microneedle patch combined with three lead zirconate titanate piezoelectric transducers (PZT) and electrochemical micro‐actuator was constructed, which was connected by a flexible circuit and encapsulated by transparent PDMS elastomer (**Figure** **9**a‐i).^[^ ^152^ ^]^ The fabricated microneedle patch exhibited a uniform arrangement and was an ideal drug carrier (Figure 9a‐ii,a‐iii). Taking advantage of ultrasound‐inducing signal propagation, PZT was received and the circuit was activated, which was beneficial for less invasive extracorporeal operation treatment. Besides, PZT could be set on top of the microneedles to develop an integrated wearable intelligent microneedle patch. Cui and coworkers designed a closed‐loop PLA microneedle system consisting of sensing microneedle, drug delivery microneedle, and ultrasonic‐based PZT. The device could be penetrated into the artificial skin with the real‐time detection, further accelerating the diffusion of insulin by ultrasound.^[^ ^153^ ^]^

The exploration of acoustic waves on the piezoelectric effect opens a new paradigm for the advancement of intelligent, remote controllable, wireless, self‐powered microneedle devices. Sound energy can be transformed into electrical signals of a certain frequency by stimulating piezoelectric materials due to the generation of resonations. Zhan and their colleagues adopted smartphones to offer acoustic source to wirelessly motivate the integrated system composed of the resonator, PZT receiver, PVP microneedle array, and neural stimulation electrodes.^[^ ^154^ ^]^ To concentrate and enlarge the acoustic energy, biforate resonant cavity structure was designed, which could effectively spread sound energy to PZT, thus favoring the generation of electrical energy (Figure 9b‐i). Besides, according to the working principle, the frequency of sound energy should be considered, which was explored from 1 to 8 kHz (Figure 9b‐ii). The results showed that when the frequency was 4450 Hz, the maximum output current signal was detected (Figure 9b‐iii). In the meanwhile, the output current was negatively related to the increase of distance between the resonant cavity and the sound source. Microneedles with a height of 400 µm and a length of 370 µm served as the drug delivery component (Figure 9biv). Evident puncture cavities with a depth of 150 µm could be observed upon the rat's skin, verifying the outstanding mechanical strength of PVP microneedles (Figure 9b‐v, b‐vi). Such acoustic‐based piezoelectric‐driven microneedles provided a remote regulation, wirelessly, miniaturized method for the advancement of self‐powered devices, which would be potential for various fields and facilitate personalized healthcare therapy.

Although piezoelectric‐driven microneedles have made great progress, there remain some challenges that would hinder their applications. In detail, to achieve an ideal piezoelectric effect, the microneedles need to be directly integrated with tissues or implanted into body, which calls for great biosafety, biodegradability, and flexibility. In addition, in terms of external stimuli, including ultrasound and acoustic waves, it is difficult to obtain a stable, accurate, effective signal output, thereby inducing a low‐level electric field helpful for tissue growth. Moreover, moisture and body fluids can influence the piezoelectric effect. Collectively, there exists a long way in the development of piezoelectric‐driven microneedles.

### 3.4 Others

Except for the above‐mentioned types, there are some reports concerning the combination of conductive‐driven types and triboelectric/piezoelectric driven types to achieve high‐performance electrical microneedles. The designed device can not only solve the supply dependance issues of conductive‐driven microneedles, but also improve the energy conversion capacity of triboelectric/piezoelectric‐driven microneedles. Ge et al. proposed a gelatin methacryloyl (GelMA) microneedle device composed of ZnO nanoparticles and PANI polymers.^[^ ^155^ ^]^ Owing to the integration of PANI polymers, the GelMA microneedle exhibited outstanding electrical conductivity. By the way, piezoelectric effect through converting mechanical motion into electrical energy imparted the GelMA microneedles with the self‐powered ability. A similar design principle was presented by Feng et al., who fabricated a self‐powered electronic microneedle by combing triboelectric effect with metal microneedles.^[^ ^156^ ^]^ Superior electrical output performance was achieved and enabled the microneedle system to obtain stable motion sensing, further beneficial for the generation of pulsed electrons from triboelectric components.

## 4 Applications in Wound Treatment

With a deeper understanding of the mechanisms regarding electrical stimulation on wound healing and in light of the significant features of the developed electrical microneedles, they play a decisive role in the field of wound treatment. In this section, the highlight is specifically focused on the potential value of electrical microneedles in the several aspects that can participate in and promote wound healing and management, including controllable drug release, real‐time monitoring, and electrical therapy.

### 4.1 Controllable Drug Release

**Figure 10:**
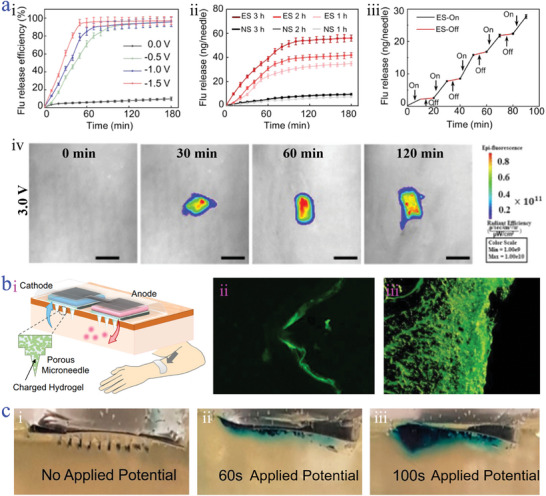
Controllable drug release. a) Drug cumulative release profiles of PLA‐Pt‐PPy microneedles under different applied electrical stimulation intensities i); drug cumulative release profiles of PLA‐Pt‐PPy microneedles at different prolonged time of applied electrical stimulation ii); drug cumulative release profiles of PLA‐Pt‐PPy microneedles on/off electrical stimulation iii); fluorescence images of rat skin treated with PLA‐Pt‐PPy microneedles under electrical stimulation of 3.0 V iv).^[^ ^157^ ^]^ Reproduced with permission: Copyright 2022, American Chemical Society. b) Schematic diagram of the PMN i); fluorescence images of pig skin treated with pure PMN ii) and modified PMN iii).^[^ ^158^ ^]^ Reproduced under terms of the CC‐BY license: Copyright 2021, Springer Nature. c) Photographs of CAP microneedles with/without electric potential punctured into the gelatin.^[^ ^159^ ^]^ Reproduced under terms of the CC‐BY license: Copyright 2019, American Chemical Society.

Controllable drug release is a crucial step for achieving effective and precise disease therapy and management, especially in addressing many traditional medication shortcomings such as weak targeted release capability, reduced drug efficacy, and inevitable side effects. Microneedles, a promising drug carrier with the advantages of minimizing invasiveness, painlessly puncture ability, and desirable self‐administration, are considered as a useful tool for realizing controllable, intelligent, and transdermal drug delivery. To further improve the efficacy of microneedles‐based drug delivery system, the combination of electrical stimulation has claimed to be an attractive option attributed to the augmented therapeutic outcomes in the synergistic effects of enhanced drug release and electrical therapy/intervention. For instance, in the study of Guo et al., effective electrostimulation could regulate drug delivery.^[^ ^157^ ^]^ To be specific, the drug release capacity of the PLA‐Pt‐PPy microneedles was dependent on the value of applied voltage and the electrostimulation time. It was observed from **Figure** **10**a‐i that the cumulative release amounts showed an upward trend with the value increase of applied voltage. And the maximum cumulative release amount could reach to 80%–90%, while the group without electrostimulation was only 10%–20%. A similar phenomenon was detected in the Figure 10a‐ii that the prolonged electrostimulation time promoted the enhancement of drug delivery. The positive influence of electrical stimulation on drug release was mainly due to the electrostatic repulsion, in which more charge transfer induced the reduced state of PPy, forcing the release of anionic drugs. Such drug release was capable of flexibly regulated through controlling the action of electrostimulation (Figure 10a‐iii). Furthermore, the in vivo electrically responsive controlled drug delivery in rat skin was achieved (Figure 10a‐iv), thereby facilitating the therapeutic efficacy of transdermal diseases. In addition, exercise‐induced TENG of microneedle array could manipulate the state of electrostatic attraction of PPy, so that controllable release of optogenetically engineered extracellular vesicles could be realized.

Apart from the mechanism of electrostatic repulsion by electrical stimulation, iontophoresis has gained extensive attention for advancing transdermal drug delivery, which is supported by electroosmotic flow (EOF) and electrophoresis. Evidence suggests that larger EOF contributes to accelerated transdermal drug transport. To achieve the goal, Nishizawa et al. synthesized an ion‐conductive porous microneedle (PMN), which was modified by a hydrogel with sulfonic groups accounting for the formation and enhancement of EOF with the anode‐to‐cathode direction (Figure 10b‐i).^[^ ^158^ ^]^ The porous structure of PMN was conducive to the drug permeation, and the low invasiveness of PMN could reduce skin resistance. Benefitting from these features, the modified PMN with EOF effect dramatically promoted the transport of drugs with large molecules (FITC‐dextran) when it was penetrated into the pig skin. The results were shown in Figure 10b‐ii that the pure PMN had a huge difficulty in drug delivery, while an evident fluorescence with deeper penetration was observed in the modified PMN (Figure 10b‐iii). This may be due to the sufficient EOF generation of the modified PMN, which greatly drove the administration of dextran.

Electrochemically controllable drug release is increasingly being heralded as an intriguing technology in drug administration. In particular, the integration of electrochemistry and pH‐responsive materials offers a promising approach for controllable drug release. A typical example was proposed by Davis’ group and the pH‐responsive cellulose acetate phthalate (CAP) microneedles were developed.^[^ ^159^ ^]^ The principle of electrochemically controlled drug release was that electric potential induced the pH variations of CAP microneedles, which resulted in their swelling and dissolvable, further facilitating the drug release. Applying electric potential with varying degrees, the condition of swelling and dissolvable was changed and the drug release could be regulated (Figure 10c‐i‐c‐iii). In addition, taking inspiration from the electrochemically controlled drug release, another conductive microneedle fabricated by PEDOT polymers was presented, which adopted the electrochemical interactions between PEDOT polymers and charged drugs to manipulate the drug release status.

### 4.2 Real‐Time Monitoring

Wound condition real‐time monitoring has been an emerging concept that can provide critical and real‐time information for guiding and managing the treatment of wound‐related complications through tracking physical and/or chemical parameters. Electrochemical sensors including numerous bioelectronics with the advantages of convenience, accuracy, and biocompatibility, have been potential candidates for diagnosis, treatment, and physiological research.^[^ ^160^, ^161^ ^]^ Although with considerable progress, there remains an urgent desire to design minimally invasive, user‐friendly bio‐sensing devices for achieving patient comfort, biosafety, and functional diversity. Fortunately, as an attractive less invasive transdermal device, microneedles show adjustable dimensions and shapes in terms of length, sharpness, and density, allowing for opening scopes in sampling, detecting, sensing as well as monitoring areas. By integrating biosensors and microneedles, various electrical microneedle‐based sensors have been employed for monitoring health/disease‐related biomarkers. Especially, along with the advancement of material science and technology engineering, microneedles with diverse structures have been fabricated, including solid, hollow, coated, etc. Electrical microneedles with these types play different roles in sensing and monitoring, which are introduced and discussed as follows.

**Figure 11:**
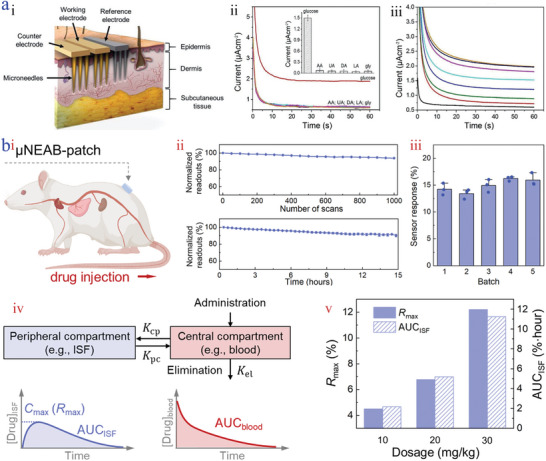
Real‐time monitoring. a) Scheme of the three‐electrode sensing patch punctured in tissue i); interference study of several groups treated with applied potential of 0.35 V ii); chronoamperometric response curve iii).^[^ ^164^ ^]^ Reproduced with permission: Copyright 2021, John Wiley and Sons. b) Schematic diagram of the µNEABs punctured into rat skin i); normalized continuous µNEABs readouts in the condition of number of scans ii) and extended operation time iii); illustration of PK measurement iv); the relationship of *R_max_* and AUC_ISF_ with the increase of tobramycin doses v).^[^ ^167^ ^]^ Reproduced with permission: Copyright 2022, AAAS.

The monitoring of blood has always been the golden standard for disease diagnosis.^[^ ^162^ ^]^ Derived from blood transcapillary filtration, skin interstitial fluid (ISF) which contains a large number of valuable biomarkers such as urea, proteins, glucose, Na^+^, K^+^, and so on, has been extensively surveyed for tracking and monitoring disease progress. Among them, glucose, an essential biomarker that is associated with diabetes, has been valued and monitored for the management and treatment of diabetic wounds.^[^ ^163^ ^]^ In general, the glucose level of a healthy person ranges from 3.6 to 6.0 mmol L^−1^, while that of patients will be disordered. To realize real‐time and minimally invasive glucose monitoring, Voelcker et al. presented a three‐electrode sensing patch made up of reference, working, and counter electrodes based on Au‐Si coated microneedles (Au‐Si‐MNA) (**Figure** **11**a‐i).^[^ ^164^ ^]^ To reduce the interference of electroactive molecules, a redox mediator (Fc‐PAMAM) was adopted and the glucose oxidase (GOx) was employed as the catalytic bioreceptor. Under the action of the applied potential of 0.35 V, the MNA with the optimized working temperature of 35 °C showed superior selectivity, great sensitivity (0.1622 µA mM^−1^ cm^−1^) and displayed a low detection limit (0.66 mM) (Figure 11a‐ii‐a‐iii). Given these features, the MNA exhibited the capability of in vivo glucose monitoring. The monitored glucose level was consistent with that in blood, indicating their applicability, which was valuable for the treatment of diabetic wounds. Besides, Park and his workers adopted a self‐powered microneedle for monitoring glucose concentration based on the glucose oxidation reaction to charge a supercapacitor.^[^ ^165^ ^]^ Taking advantage of the device, a wide range of glucose monitoring could be achieved. In addition, the hollow microneedles are not only beneficial for providing sufficient room for the fixation of the electrodes, but also conducive to drug loading and oxygen accessibility. A hollow stainless‐teel microneedle was fabricated for continuous glucose monitoring.^[^ ^166^ ^]^ Results showed that the glucose concentration was relevant to the response current/voltage of the microneedles.

Improper drug therapy or dosing will lead to toxicity and drug resistance, delaying healing time. Thus, therapeutic drug monitoring is essential for precision and personalized medicine, which shows enormous potential in the guidance of timely drug administration, thus promoting wound treatment. Emaminejad et al. proposed an electrochemical aptamer biosensing platform based on microneedles (µNEABs) (Figure 11b‐i).^[^ ^167^ ^]^ The electrically measurable signal was detected by coupling aptamer labeled with redox reporter and target (targeting vancomycin, doxorubicin, and thrombin), which exhibited long‐term stability and reproducibility (Figure 11b‐ii–b‐iii). Such electrical signal generation originated from electron transfer between the redox reporter and electrodes. Through monitoring pharmacokinetic (PK) characteristics regarding area under the curve (AUC) and exploring the relationship between AUC_blood_ and the measured µNEABs readings (AUC_ISF_), drug dosing could be guided (Figure 11b‐iv). Encouragingly, the patients’ total drug exposure for precision dosing allowed for prediction with the aid of the maximal value of the sensor response (*R_max_*) (Figure 11b‐v). A similar design principle was used on the stainless‐steel microneedles that were capable of monitoring vancomycin in the clinical range with the accuracy of 3% and the mean calculated response of 29 µm in the condition of 30 µm target.

**Figure 12:**
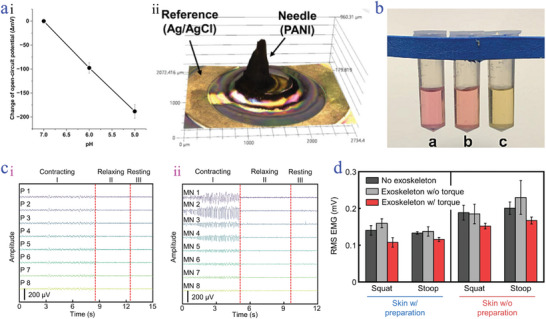
Real‐time monitoring. a) pH with the variation of open‐circuit potential; i); 3D microscope of the microneedle ii).^[^ ^169^ ^]^ Reproduced with permission: Copyright 2021, AAAS. b) Photograph of three groups (a: GOx, b: γ‐PGA‐MXene microneedle loaded with GOx, c: methyl red) after reaction with glucose with the addition of methyl red.^[^ ^171^ ^]^ Reproduced with permission: Copyright 2024, Elsevier. c) Intramuscular EMG and surface EMG signals recorded from the planer i) and electrical microneedles ii).^[^ ^173^ ^]^ Reproduced with permission: Copyright 2024, AAAS. d) EMG signals from the skin in cases of pretreated and non‐pretreated with perspiring.^[^ ^174^ ^]^ Reproduced with permission: Copyright 2024, AAAS.

Evaluation of wound status is of significance to an individual's health. pH is demonstrated as a real‐time indicator to manifest the wound condition and pH of healthy skins has distinct differences compared with that of wound skins.^[^ ^168^ ^]^ In specific, the pH above 7 was detected in the infected wound, while the healthy skin showed a pH value ranging from 4 to 6.3. Given this, the monitoring of pH plays a dominant role in reflecting the degree of wound infection, facilitating timely drug administration and therapy. Bae and his colleagues presented a pH sensor, which integrated a flexible electronic system on the microneedle array's substrate.^[^ ^169^ ^]^ The sensor exhibited a great sensitivity of 94 mV/pH (**Figure** **12**a‐i), which was derived from PANI with porous structures (Figure 12a‐ii). Besides, recent research raised by Crespo adopted a pH sensor based on stainless‐steel microneedles integrated with electrodes for realizing accurate (<1%) and precise (<2%) pH monitoring.^[^ ^170^ ^]^ Notably, the conductive microneedles combined with pH‐sensitive materials/polymers facilitate the pH monitoring and the regulation of wound microenvironment. For instance, Wang et al. used a poly (γ‐glutamic acid) (γ‐PGA) MXene microneedle loaded with GOx (a well‐known enzyme for regulating pH of microenvironment) for promoting wound healing.^[^ ^171^ ^]^ γ‐PGA and MXene could neutralize H_2_O_2_ generated from the reaction between GOx and glucose. The pH variations during the process were detected by methyl red (acid‐base indicator), which displayed a pink color, indicating the decrease of pH compared with pure glucose solution with a yellow color (Figure 12b). The regulation of an alkaline environment was helpful for inhibiting the growth of bacteria, thus favoring chronic wound healing.

Furthermore, the monitoring of electrophysiological parameters such as electrocardiography (ECG), electromyography (EMG), electroencephalogram (EEG), etc., offers a powerful route for wound management in terms of timely wound treatment and diagnosis.^[^ ^172^ ^]^ The combination of the electrical part and microneedles provides promising opportunities for obtaining valuable information of interior regions in a minimally invasive manner. Different from the planer monitoring of skin surface with relatively weak and inapparent EMG signals, evident and intensive EGM signals of muscle tissues were detected based on the fabricated electrical microneedles by Zhao's group (Figure 12c‐i–cii).^[^ ^173^ ^]^ Through the measured signals, the contracting and relaxing condition of muscle tissues could be easily judged. This distinctive feature made them an advanced system for real‐time monitoring and sensing of biological tissues, which was valuable for providing bright guidance for disease treatment. The sensing stability and accuracy are critical for real‐time monitoring. To achieve the goal, electrically conductive adhesion was employed into the electrical microneedles, which not only offered extra conductive path for improving sensing ability, but also facilitated the robust contact with skin. Taking advantage of the system, more reliable and long‐term electrophysiological signals were acquired (Figure 12d).^[^ ^174^ ^]^ Additionally, O'Mahony et al. introduced textile‐based materials as a rear backing layer into electrical microneedles to improve signal quality.^[^ ^175^ ^]^ It was demonstrated that the platform could detect EGC and EMG signals with a low signal‐to‐noise ratio.

### 4.3 Electrical Therapy

**Figure 13:**
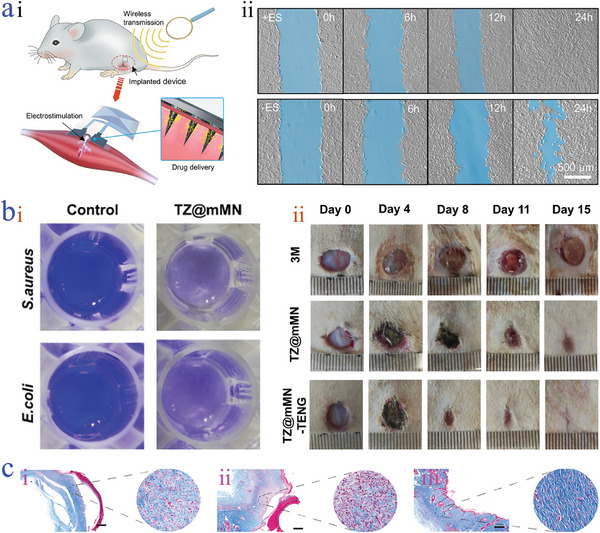
Electrical therapy. a) Schematic diagram of IBMN used as the implanted device; i); scratch wound healing assay of IBMN with/without electrical stimulation ii).^[^ ^178^ ^]^ Reproduced with permission: Copyright 2022, American Chemical Society. b) Antibacterial biofilm capacity of the TZ@mMN i); photographs of wound closure condition of three groups ii).^[^ ^155^ ^]^ Reproduced with permission: Copyright 2024, John Wiley and Sons. c) Masson's trichrome staining of the healing wound tissues on day 21 of three groups including the control group i), blank‐microneedle group ii), and microneedle with microcurrents group iii).^[^ ^185^ ^]^ Reproduced with permission: Copyright 2023, AAAS.

Biochemical cues are widely bound in tissues and cells of natural creatures for protecting and regulating them in a healthy state, while biophysical signals are responsible for the behaviors of cells including proliferation and differentiation, tissues and organs regeneration, and embryo development. Great attempts have been dedicated to exploring these biophysical signals and stimulating them to achieve the desired therapy purpose.^[^ ^176^, ^177^ ^]^ Chronic wounds, characterized by long‐term inflammation and delayed healing, have caused increased healthcare costs. When the wounds are generated, abnormal biophysical signals will appear, involving the disordered electric fields around wounds. Even worse, the trend of their collapse has side effects against cell behaviors, severing the wounds. Electrical stimulation has emerged and acted as a viable strategy for participating in controlling endogenous cell behaviors. It is worth mentioning that invasive electrostimulation based on microneedles is advantageous for acquiring a greater concentration and parallel distribution of electric field, thus promoting tissue repair. For example, Yu et al. put forward a metal (Mg)‐coated poly(lactic‐co‐glycolic acid) (PLGA) bioresorbable microneedle (IBMN) that combined the drugs and electrostimulation (**Figure** **13**a‐i).^[^ ^178^ ^]^ Through rat muscle injury model experiment, they demonstrated that electrical stimulation had a positive impact on the migration of myoblasts (Figure 13a‐ii), which was beneficial to the formation of newly regenerated myofibers compared with the untreated group. In addition, the electric field favored the reduction of inflammation and generation the collagen, accelerating the wound healing process. Similarly, Zhao's group also verified the regulation ability of LM‐encapsulated microneedles in the proliferation and migration of fibroblasts when exposed to electric field (2 V cm^−1^) for 20 h.^[^ ^110^ ^]^ Notably, Cui et al. believed that electrical stimulation derived from conductive microneedles not only facilitated cell proliferation, but also achieved organelle localization regulation.^[^ ^179^ ^]^

Bacterial infection is a huge threat to wound therapy. Recently, electrical stimulation has shown guiding significance to the antibacterial treatment. In an interesting investigation, taking advantage of the penetration ability of microneedles, bacterial biofilm could be destroyed (Figure 13b‐i).^[^ ^155^ ^]^ Accompanied by the electric field transferring to deeper tissues, the proliferation and migration of cells were controlled by the self‐reported TENG named TZ@mMN‐TENG, showing the critical role in promoting diabetic wound healing (Figure 13b‐ii). At this time, they found that electrical stimulation had a positive impact on the antimicrobial effect. The antibacterial mechanism may be ascribed to the recruitment effect induced by electrical stimulation on neutrophils and macrophages. Besides, other studies supposed that electrical stimulation could influence bacterial adhesion, thereby inhibiting the formation of bacterial biofilm.^[^ ^180^ ^]^ Despite the advantages, the antibacterial effect of single electrical stimulation remains unsatisfactory, which calls for the help of additional antibacterial agents. Jiang et al. employed electrical stimulation from the TENG microneedle system, along with the enhanced drug release, to activate the cell mobility and augment the antibacterial effect.^[^ ^181^ ^]^

Scar, a pathologic fibrosis disease, mainly appears in the remodeling stage of wound healing, which seriously affects the appearance of patients, and even causes loss of limb mobility. Some acknowledged claims thought that the formation of scars is due to the overgrowth of fibroblasts and redundant deposition of ECM.^[^ ^182^, ^183^ ^]^ Many researchers reveal that such a disordered condition is because the weakening and loss of bioelectricity cause gene dysregulation and cascading downregulation of wounds, which eventually leads to the generation of scar.^[^ ^184^ ^]^ Given this, electrical microneedles have been reported to effectively prevent scar formation via synergistic effects, including stimulation and amplifying endogenous bioelectricity, as well as effective drug delivery. For instance, Fan and his colleagues proposed a self‐reported microneedle (ZGH‐MN) with continuous and stable current output and antibacterial drug loading (zeolite imidazolate framework (ZIF‐8)).^[^ ^185^ ^]^ The involvement of the electric field contributed to the regular arrangement of collagen fibers and collagen proportion was similar to the normal skin (Figure 13c), proving the positive effect of ZGM‐MN on remolding of ECM and scar prevention. It was worth mentioning that a recent study revealed that the microhole array established by microneedles could affect and adjust the biomechanics and ultrastructure of the tissue, which was further beneficial for scar‐free wound healing.

## 5 Conclusions and Outlook

The past decades have brought tremendous development in the evolution of wound dressings with an in‐depth understanding of wound healing and advancement in fabrication technologies. Electrical stimulation has provided a significant approach via stimulating and amplifying endogenous electric field for wound therapy. In this review, we have given an introduction in aspects of the generation and function of endogenous electric field, and the working mechanism of electrical stimulation on wound healing. To achieve invasive electrical stimulation to further improve wound therapeutic effect, electrical microneedles have emerged and recent research concerning three kinds of electrical microneedles has been summarized. Specific attention was paid to their features and working principles. These electrical microneedles have offered a sea of opportunities to realize ideal wound treatments with respect to controllable drug release, real‐time monitoring, and electrical therapy. Despite the on‐going development and great promise, the reality and challenges should be considered and addressed for real‐world use, as well as future perspectives should be recognized for the next‐generation electrical microneedles.

First, it has been a daunting task to form a unified standard of applied electrical stimulation. As the existence of differences in individual, wound size and type, there remains huge difficulty in evaluating and confirming effective electrical stimulation for wound healing. Efforts need to be dedicated to the systematic study of electric field on different individuals and wound conditions involving area, depth, cause, etc., accompanied by methods and analysis of cytology and biology. Besides, regarding the distinctive feature of three kinds of electrical microneedles, it is necessary to investigate their applied intensity, frequency, and duration time according to the working principle difference between electrical stimulation and wound types.

The second issue refers to the exploration of electrical microneedles. Although many studies have been reported, the research on electrical microneedles is still in the preliminary stages, even less for wound healing. In specific, the complicated and varied wound surface morphology poses huge difficulties for puncturing. Also, electrical microneedles with a limited electrical stimulation area restrict drug loading capacity, which has an insufficient therapeutic effect when comes to large‐scale wounds. In addition, the trouble in achieving good electrical stability and reliability also hinders the development of electrical microneedles. Moreover, the biological effects and long‐term biosafety relating to electrical microneedles and the applied electrical stimulation still need to be considered and further assessed. These challenges call for the design of electrical microneedles with high requirements and superior performance. Directions can be converted to advancements in manufacturing and material science for the production of large‐scale, cost‐effective, and bioaffinity electrical microneedles. Along with the integration of a smarter system with standard calibration procedures, programmed drug delivery, rational electrical parameter regulation, and nanotechnology in biosensing, the desired electrical microneedles with good penetration ability, effective drug treatment, as well as sensitive, accurate, and biosafety electrical stimulation are expected to be realized.

The third issue concerns the clinical treatment of skin wounds. Electrical microneedles for clinical therapy have a long way to go due to the following several drawbacks. In general, it is still a great challenge for keeping biosafety involving electrical stimulation in practical applications, which may be related to risk assessment, intricate regulatory authorities, and ethical tissues. Besides, the fixation of microneedles in complex and dynamic physiological environments is another urgent problem that needs to be solved. More importantly, the immunological response as well as biodegradation property of biomaterials for electrical microneedles call for more attention, which are significant points for clinical applications. Faced with these challenges, the direction of future research is expected to achieve interdisciplinary intersection and collaboration in aspects of material science, tissue engineering, flexible electronics, and clinical medicine, thus promoting the advancement of wound treatment.

Collectively, although some shortcomings are being unaddressed, great achievements have been made of electrical microneedles, which show great promise in accelerating wound healing. We do believe that this review will give researchers a comprehensive understanding of electrical microneedles and offer more inspiration for promoting the development of next‐generation electrical microneedles.

## Conflict of Interest

The authors declare no conflict of interest.

## Author Contributions

Y.J.Z. conceived the idea; Y.W. wrote the manuscript; Y.W., L.J.C., L.F., L.W., F.K.B. and W.J.S. revised the manuscript.
